# Highly Sensitive Measurement of the Refractive Index of Mesoporous Hollow Silica Microcapsules Using Whispering Gallery Mode Resonances

**DOI:** 10.3390/s26010250

**Published:** 2025-12-31

**Authors:** Qisheng Xu, Sadok Kouz, Aatir Khan, Naheed Hossain, Nizar Bchellaoui, Abdel I. El Abed

**Affiliations:** Laboratoire Lumière Matière et Interfaces (LUMIN), UMR 9024, Ecole Normale Supérieure Paris Saclay, CentraleSupélec, CNRS, Université Paris-Saclay, 4 Avenue des Sciences, 91190 Gif-sur-Yvette, France; qisheng.xu@ens-paris-saclay.fr (Q.X.); sadok.kouz@ens-paris-saclay.fr (S.K.); aatir.khan@ens-paris-saclay.fr (A.K.); nhossain@ens-paris-saclay.fr (N.H.); nizar.b.fsm@gmail.com (N.B.)

**Keywords:** mesoporous silica, microfluidics, whispering gallery modes, effective refractive index, optical resonances, refractometry, fourier transform spectroscopy

## Abstract

Monodisperse mesoporous hollow silica microcapsules present unique opportunities for advanced optical characterization due to their tunable nanostructure, high porosity and easy functionalization. A critical and challenging parameter in the optimization of these applications is the accurate determination of the effective refractive index, which governs light propagation and confinement within the nanostructured matrix of such mesoporous materials. In this study, individual mesoporous hollow silica microcapsules doped with Rhodamine B dye were analysed optically by exploiting whispering gallery mode (WGM) resonances, enabling non-destructive, single-particle refractometry with nanostructural sensitivity. Fourier Transform analysis of the fluorescence emission spectra revealed sharply defined, periodically spaced WGM peaks. For microcapsules with an 88 μm diameter, the measured intermodal spacing (Δλ = 1.296 nm) yielded an effective refractive index of 1.164. The measured value of the effective refractive index was cross-validated using Lorenz–Lorentz and Bruggeman effective medium models, both predicting porosity values (~63%) that closely match independent Brunauer–Emmett–Teller (BET) nitrogen adsorption measurements. The excellent agreement between optical and adsorption-based porosity demonstrates that WGM spectroscopy combined with Fourier analysis is a powerful, label-free, and non-invasive technique for correlating nanoscale porosity with macroscopic optical properties. This approach is widely applicable to single-particle analyses of nanostructured dielectric materials and opens new possibilities for in situ optical metrology in the development of advanced photonic, catalytic, and biomedical platforms.

## 1. Introduction

Mesoporous silica materials represent an exceptional class of nanostructured dielectrics that have fundamentally transformed numerous scientific and technological domains over the past three decades [1,2,3,4,5,6,7,8,9]. Characterized by an ordered porous architecture with precisely controlled pore sizes ranging from 2 to 50 nm, these materials exhibit a unique combination of properties, including exceptionally high specific surface areas (often exceeding 500 m^2^/g), remarkable thermal and chemical stability, tunable pore geometries, and exceptional biocompatibility and functionalization. These characteristics have established mesoporous silica as an indispensable platform for diverse applications spanning catalysis, drug delivery systems, gas storage and separation, environmental remediation, biosensing, and advanced photonic devices [10,11,12].

The controlled synthesis of mesoporous silica through microfluidic templating approaches enables the fabrication of smart structures with defined spherical geometries and hierarchical porous architectures [13,14,15], thereby opening unprecedented avenues for sophisticated optical applications [16,17,18,19,20]. Such well-shaped microspheres may be used as optical microresonators capable of confining light through total internal reflection and whispering gallery modes (WGMs). The latter represents electromagnetic field distributions that circulate around the perimeter of the resonator with minimal loss due to constructive interference [19,20,21,22]. The extraordinary sensitivity of WGMs to variations in the effective refractive index ($n_{\mathrm{eff}}$), which arises from the extended photon lifetime and enhanced light–matter interaction length within the resonator cavity [23,24,25], combined with the capability to achieve high-quality factors in well-shaped spherical microspheres, makes WGM resonators powerful tools for label-free biosensing and non-invasive analysis of material properties at the microscopic scale [22]. Recent advances have demonstrated their applicability across a broad spectrum of sensing modalities with unprecedented sensitivity [26].

A critical and challenging parameter in the optimization of these applications is the accurate determination of the effective refractive index, which governs light propagation and confinement within the nanostructured matrix of these mesoporous materials. Precise knowledge of this property is essential for the rational design of advanced optical coatings, photonic crystals, and sensing platforms [27]. However, unlike dense materials, the effective refractive index of mesoporous materials is a complex composite property that depends on multiple interdependent parameters: (i) the overall porosity and pore volume fraction, (ii) the geometry and connectivity of the porous network (cylindrical, spherical, bicontinuous, or hierarchically interconnected), (iii) the pore size distribution and surface roughness characteristics, (iv) the chemical composition and surface functionalization of the pore walls, and (v) the nature of the medium filling the porous voids (air, water, organic solvents, or specific analyte molecules) [28,29,30]. This multi-parametric dependence makes its measurement significantly more complex than for traditional dense materials, necessitating specialized techniques and sophisticated effective medium approximations (EMAs) to accurately determine the optical properties of these hierarchically nanostructured systems [31,32].

Traditional bulk characterization methods such as ellipsometry, Abbe refractometry, and prism coupling often fail to provide accurate measurements for highly porous materials due to scattering losses, surface roughness effects, and the difficulty in preparing suitable sample geometries. To model and predict $n_{\mathrm{eff}}$, various effective medium approximations (EMAs) are employed, most notably the Bruggeman approximation (for symmetric, interconnected mixtures) and the Lorenz–Lorentz model (for dilute systems with spherical inclusions) [28,29]. The accuracy of these models depends critically on their underlying assumptions about pore geometry and requires careful validation against experimental measurements [33].

We developed, in a previous work, a droplet-based microfluidic approach for fabricating highly monodisperse mesoporous hollow silica microcapsules featuring radially ordered mesoporous shells [13,34]. This method provides exquisite control over microcapsules morphology, size distribution, and porous architecture, producing spherical structures with exceptional optical quality.

We demonstrate in this study that these microfluidic-synthesized mesoporous hollow silica microcapsules can act as high-quality WGM microresonators. By incorporating Rhodamine B dye into the silica shell and applying Fourier Transform (FT) analysis of the fluorescence spectra, we extract the effective refractive index of the shell material with high precision at the single-particle level. We compare the experimentally obtained $n_{\mathrm{eff}}$ to theoretical predictions from the Lorenz–Lorentz and Bruggeman effective medium models and benchmark the optically inferred porosity against nitrogen adsorption measurements performed via the Brunauer–Emmett–Teller (BET) method. Our results establish a direct link between nanoscale porosity and macroscopic optical behavior, confirming that WGM spectroscopy offers a robust, non-destructive, and highly sensitive refractometric tool for the characterization of advanced photonic materials.

## 2. Experimental Section

### 2.1. Microcapsules Fabrication Procedure

**Figure 1:**
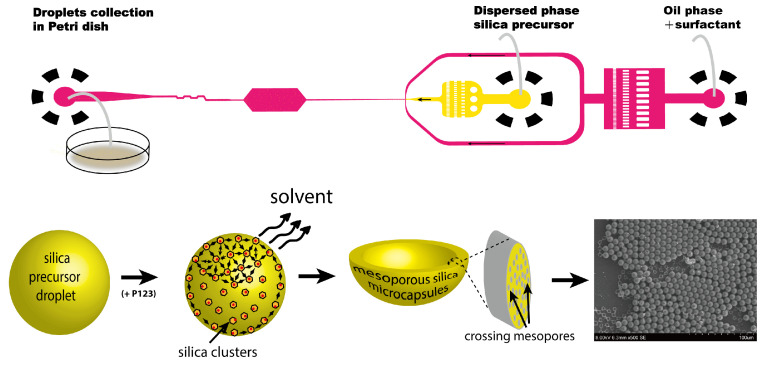
Schematic of the microfluidic approach used for droplet-based synthesis of mesoporous hollow silica microcapsules.

The synthesis of monodisperse mesoporous hollow silica microcapsules was performed using a custom droplet-based microfluidic platform adapted from our previous work [13,34]. As shown in Figure 1, the complete fabrication process involves three key stages: silica precursor microdroplet production, collection, and microcapsule solidification. A highly monodisperse emulsion was first generated at the flow-focusing junction with precise flow rate control ($Q_{\mathrm{inner}}\;=\;100\;\mu L/\min$, $Q_{\mathrm{outer}}\;=\;500\;\mu L/\min$). The collected emulsion then underwent ambient gelation for 12–24 h, followed by controlled drying at 25 °C and 40% relative humidity for 48 h. The dispersed phase contained a sol–gel precursor mixture of tetraethyl orthosilicate (TEOS) as the silica source, a 1:1 (*v*/*v*) ethanol/water solvent system, 0.1 M HCl catalyst, and 4 wt% Pluronic^®^ P123 (EO_20_-PO_70_-EO_20_) as the mesopore template. This protocol yielded microcapsules with controlled size and shell thicknesses exhibited excellent monodispersity (polydispersity index < 5%), as detailed in Section 3.1. SEM analysis revealed a spherical uniformity greater than 95%, while optical characterization demonstrated excellent visible-light transparency and a stable whispering gallery mode, as detailed in Section 3.2. These qualities, combined with the precisely controlled mesoporous architecture, made the microcapsules ideal for optical interrogation and refractive index measurements.

The microfluidic chips used for droplet generation were fabricated using standard soft lithography techniques. First, a master mold was created by patterning SU-8 photoresist on silicon wafers, followed by replication with PDMS (Sylgard 184) cured at 70 °C for 4 h. The cured PDMS was then bonded to glass substrates using oxygen plasma treatment. The final device architecture featured one dispersed-phase inlet for the silica precursor solution and two continuous-phase inlets for carrier fluids, converging at an optimized flow-focusing junction designed for monodisperse droplet generation. A commercially available HFE 7500 fluorinated oil (3-ethoxy-dodecafluoro-2-trifluoromethyl-hexane, (Inventec, Bry-sur-Marne, France), having a density of 1.62 g/cm^3^, was used as the carrier oil. This oil does not cause PDMS swelling and does not solubilize most non-fluorinated organic molecules, including droplet contents. Droplets were stabilized using a home-prepared copolymer surfactant, derived from a commercially available carboxy-terminated fluorinated polymer, namely Krytox 157-FSH^®^ (Dupont, Paris, France) and a solution of benzyl-trimethylammonium hydroxide (BTA, Sigma-Aldrich, Saint-Quentin-Fallavier, France).

To enable optical interrogation via fluorescence spectroscopy, Rhodamine B dye was introduced into the mesoporous hollow silica microcapsules. Rhodamine B is a cationic fluorophore with a high quantum yield and strong absorption in the green spectral range, making it an ideal probe for whispering gallery mode (WGM) excitation in the visible domain. Its small molecular size facilitates diffusion into mesoporous silica networks [35]. Two loading strategies were evaluated:

1. In situ doping, in which Rhodamine B was directly added to the sol–gel precursor prior to droplet formation. While this approach allowed for dye entrapment during silica shell formation, it occasionally led to fluorescence self-quenching and a non-uniform spatial distribution due to uncontrolled encapsulation kinetics.
2. Post-synthesis adsorption, where fully dried capsules were immersed in a 10 μM aqueous Rhodamine B solution for 24–48 h. In this method, dye molecules gradually diffused into the mesoporous framework and adsorbed onto the internal pore walls via electrostatic and van der Waals interactions. After loading, the capsules were rinsed thoroughly under vacuum to remove unbound dye.

**Figure 2:**
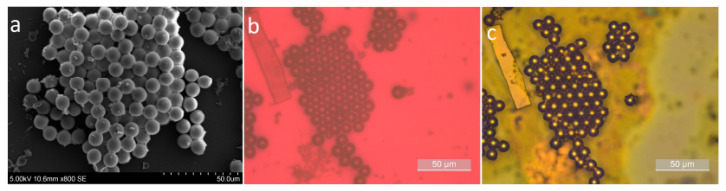
(**a**) Surface scanning electron microscopy (SEM) image of mesoporous hollow silica microcapsules prior to dye loading, confirming uniform spherical morphology and intact shell structure, (**b**,**c**) optical microscopy images of microcapsules before (t = 0 h) and after (t = 24 h) immersion in Rhodamine B solution.

The post-synthesis method was selected for all optical experiments in this work, as it yielded a stronger and more uniform fluorescence signal, with better reproducibility across samples. Bright field microscopy revealed progressive swelling of the capsules during immersion, particularly in samples with thinner shells, indicating significant dye uptake. Scanning electron microscopy (SEM) before dye loading (Figure 2) confirmed the intact spherical morphology, while optical microscopy after 24 h of immersion showed an increase in diameter due to dye infiltration. Fluorescence microscopy after 48 h confirmed strong, shell-localized emission, validating successful dye loading into the mesoporous matrix.

### 2.2. Optical Characterization and Refractive Index Measurement

**Figure 3:**
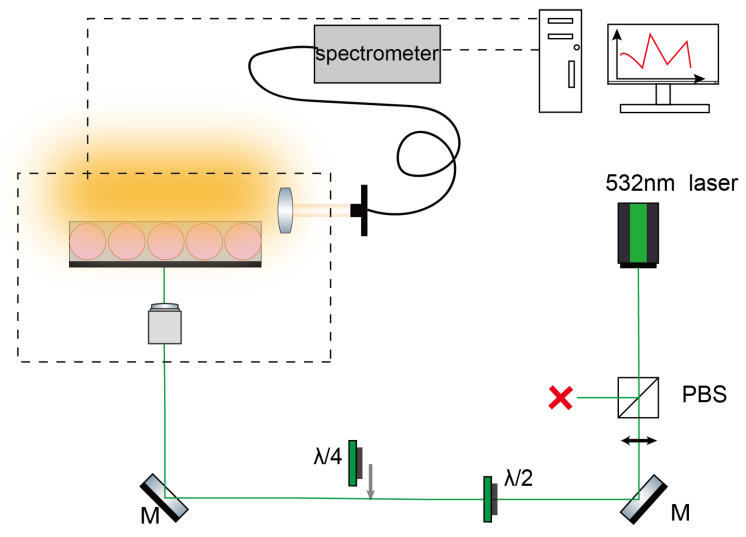
Schematic of the optical setup used for WGM fluorescence spectroscopy. Excitation and collection are performed using orthogonal objectives to enhance equatorial mode resolution.

The optical setup used to record the whispering gallery mode (WGM) spectra is presented in Figure 3. The WGMs emitted by individual dye-loaded microdroplets were observed using a free-space setup specifically optimized for spectrally resolved detection of laser-induced fluorescence. Excitation was provided by a frequency-doubled Nd:YAG pulsed laser (λ = 532 nm, 500 ps pulse width, 10 Hz repetition rate). The beam was directed normal to the microfluidic plane and focused onto a selected droplet with a 10× microscope objective. Before reaching the sample, the laser beam was expanded to a diameter of roughly 100 μm in order to illuminate the whole droplet cross section and ensure uniform pumping. The pulse energy after attenuation was set to approximately 6 μJ, slightly above the threshold required to observe stimulated emission within the droplet. The emitted light was collected in the equatorial plane by a lens positioned about 18 cm from the microdroplet. The collected signal was coupled into a multimode fiber and sent to a grating spectrometer (Acton Spectra Pro 2500i, Teledyne, France), equipped with a thermoelectrically cooled CCD detector (PIXIS 110B, (Princeton Instruments, Teledyne, France). For each spectrum, the signal corresponding to ten successive laser shots was integrated to improve the signal-to-noise ratio. The spectrometer was operated with a 1200 grooves/mm grating and a 50 µm entrance slit, providing an effective spectral resolution of approximately 0.1 nm in the 600–700 nm range, which is significantly smaller than the measured free spectral range of the WGMs (1.296 nm). This configuration ensures that individual resonances are spectrally resolved and that the intermodal spacing can be determined with high accuracy. A separate imaging arm was used to monitor the droplet and the excitation region. A CMOS camera (UI324xCP-C, IDS Imaging France) fitted with a Navitar 6000 zoom objective provided real-time, high-magnification visualization, allowing verification of the beam alignment and detection of any droplet motion.

Whispering gallery modes appeared as sharp, periodically spaced peaks superimposed on the broadband Rhodamine fluorescence background. To enhance spectral resolution, emission was collected near the equator of the capsule, where the light experiences maximal confinement and longest path length. Spectral analysis was performed using Lorentzian multi-peak fitting and Fourier Transform (FT) processing. Prior to transformation, a Hann window function was applied to the normalized spectrum to suppress edge effects. The intermodal spacing Δλ was extracted from the FT amplitude spectrum, and the optical path length was calculated as:

$$l_{\mathrm{opt}}\;=\;\frac{\lambda^{2}}{\Delta \lambda }$$

Given the capsule radius *R*, measured via calibrated optical microscopy, the effective refractive index was calculated using:

$$n_{\mathrm{eff}}\;=\;\frac{l_{\mathrm{opt}}}{2\pi R}$$

A detailed presentation of the WGM-derived refractive index, including the extracted values, associated uncertainties, and their physical interpretation, is provided in Section 3.3.

### 2.3. Porosity Characterization via BET Nitrogen Adsorption

The porosity of the mesoporous hollow silica microcapsules was characterized by nitrogen adsorption–desorption measurements at 77 K using a Micromeritics FlowSorb II 2300 surface area analyzer (Brunauer–Emmett–Teller method). Prior to analysis, the microcapsules were gently crushed into a fine powder and degassed at 200 °C under vacuum for 12 h to ensure full accessibility of the internal pore network and removal of physisorbed species.

The specific surface area was determined from the adsorption branch in the relative pressure range $0.05<P/P_{0}<0.30$, where the BET equation is valid and the corresponding plot exhibited excellent linearity ($R^{2}>0.999$). The total pore volume $V_{\mathrm{pores}}\;=\;0.76\;{\mathrm{cm}}^{3}/g$ was obtained from the adsorbed amount at $P/P_{0}\;\approx \;0.99$ using the Barrett–Joyner–Halenda (BJH) model applied to the adsorption branch. The pore-size distribution, also derived from the BJH analysis, was centered around 5.9nm, confirming the mesoporous structure templated by Pluronic^®^ P123 [13].

The effective porosity (void fraction) ϕ of the silica shell was calculated from the pore volume and the skeletal density of silica $\rho_{{\mathrm{SiO}}_{2}}\;=\;2.22\;{g/\mathrm{cm}}^{3}$ via:

$$\phi \;=\;\frac{V_{\mathrm{pores}}}{V_{\mathrm{pores}}+1/\rho_{{\mathrm{SiO}}_{2}}}\;=\;\frac{0.76}{0.76+0.45}\;\approx \;0.63\;\left(63\%\right).$$

This protocol follows the same methodology applied in our previous study of the same material system [13], which reported a specific surface area of $514\;m^{2}/g$, a pore volume of $0.76\;{\mathrm{cm}}^{3}/g$, and an average pore diameter of 5.9nm. In the present work, the BET-derived porosity serves as an independent structural benchmark for comparison with the optically extracted effective refractive index; it is not used as an input parameter for the whispering-gallery-mode analysis.

## 3. Results and Discussion

### 3.1. Microfluidic Synthesis of Mesoporous Hollow Silica Microcapsules with Different Sizes

**Figure 4:**
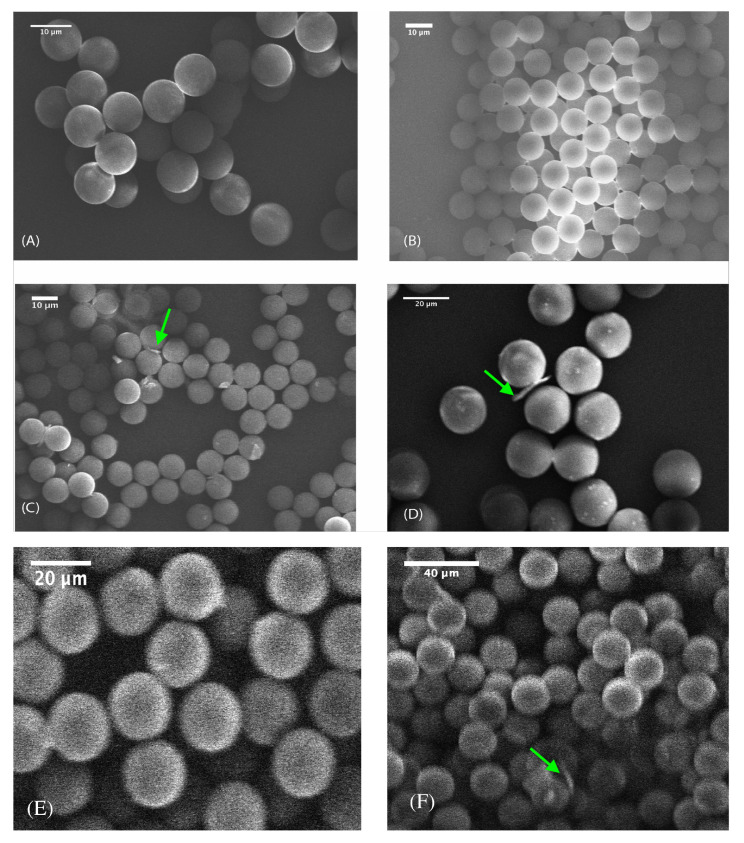
SEM images showing the change of hollow silica microcapsules size obtained when using droplets for different sizes: 30 μm (**A**); 50 μm (**B**); 70 μm (**C**); 90 μm (**D**); 120 μm (**E**) and 130 μm (**F**). Arrows show the debris of microcapsules shells which enabled to measure the effective shell thickness of microcapsules.

In our previous work [13,34], we have mainly focused on the synthesis of highly monodisperse microcapsules with a diameter of 10 μm, produced from droplets with a diameter of about 30 μm and a silica precursor solution concentration equal to 0.34 M. Due to the expected small refractive index contrast between the mesoporous shell of hollow silica microcapsules (1.16) and the outer medium’s (air), we target also the synthesis and optical investigations of larger hollow silica microcapsules in the present work. Figure 4 and Table 1 show, respectively, the change in silica microsphere size versus initial droplet sizes ranging from 30 μm to 130 μm.

It is interesting to note that the size of the microspheres increases in a stepwise manner by regards to the droplet size within the investigated range of droplet sizes while using the same concentration of TEOS (0.34 M) for all droplets.

The experimental shell thickness shown in Table 1 was determined by measuring the thickness of debris from broken microcapsules (see Figure 4), where the shell unfolds into thin sheets. Measuring the thickness of these sheets using SEM microscopy provides a direct estimate of the shell thickness.

The shell thickness can be calculated Theoretically by considering the silica precursor concentration, the initial droplet volume, and the density of the dried silica material. First, the volume of the liquid droplet is determined by assuming a spherical shape:

$$V_{g}\;=\;\frac{4}{3}\pi {\left(\frac{d_{g}}{2}\right)}^{3}$$

where $d_{g}$ is the diameter of the initial liquid droplet. This volume is converted from cubic micrometers to liters to align units with the precursor concentration, *C*, expressed in moles per liter.

Next, the total mass of silica in the droplet is calculated by multiplying the molar concentration of the silica precursor by its molar mass and the droplet volume:

$$m_{{\mathrm{SiO}}_{2}}\;=\;C\times M_{\mathrm{TEOS}}\times V_{g}$$

where $M_{\mathrm{TEOS}}$ is the molar mass of the tetraethyl orthosilicate precursor.

Assuming that all the silica in the precursor condenses to form a spherical shell around a hollow core, the shell thickness *h* can be deduced from mass conservation. The shell volume equals the mass divided by the density ρ of solid silica, and the shell surface area is approximated by the outer spherical surface:

$$V_{\mathrm{shell}}\;=\;\frac{m_{{\mathrm{SiO}}_{2}}}{\rho },\;A\;=\;4\pi r_{p}^{2}$$

where $r_{p}$ is the radius of the final dried particle. The shell thickness is then given by the shell volume divided by the surface area: $h\;=\;\frac{V_{\mathrm{shell}}}{A}\;=\;\frac{m_{{\mathrm{SiO}}_{2}}}{4\pi r_{p}^{2}\rho }$. This formula assumes a uniform, continuous shell thickness and neglects any porosity or irregularities. However, experimental observations reveal that the droplet does not always condense into a single, uniform microsphere. Instead, when the silica mass encapsulated in a droplet surpasses the physical constraints imposed by the Laplace pressure and material density and achievable shell thickness, the droplet undergoes division into multiple smaller spheres. This phenomenon occurs because forming a single particle with an excessively thick shell is energetically unfavourable or even physically impossible.

**Figure 5:**
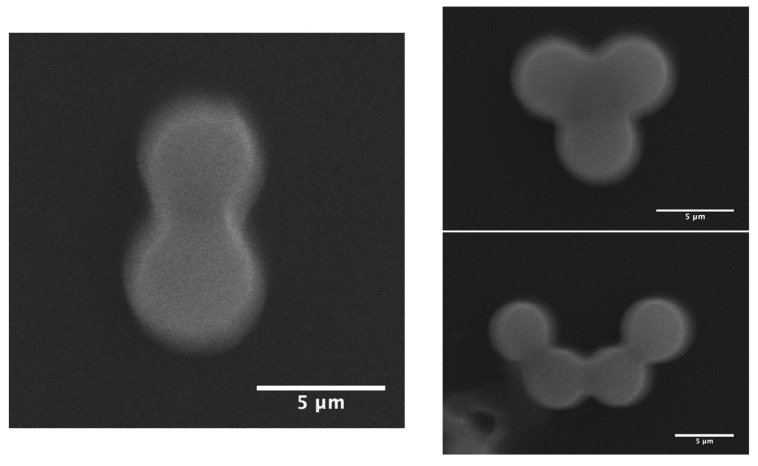
SEM image capturing a droplet in the process of dividing into multiple smaller daughters microspheres during microfluidic synthesis. The division occurs when the combined silica mass exceeds the capability of a single stable shell, leading to droplet splitting. This phenomenon supports the theoretical model explaining the relationship between silica concentration, shell thickness, and particle number.

Our model captures this behaviour by introducing the integer *n*, which represents the number of smaller spheres formed from one original droplet. By dividing the total silica mass by *n*, the model effectively redistributes the silica material among multiple spheres, resulting in a reduced shell thickness for each. This iterative approach enables accurate reconciliation between the experimentally observed shell thickness and particle size distributions and the predictions from first principles according to $h\;=\;\frac{m_{{\mathrm{SiO}}_{2}}/n}{4\pi r_{p}^{2}\rho }$ relationship. The model does not only quantifies the shell thickness but also inherently explains the droplet splitting process as a natural mechanism to maintain physically realistic shell densities and geometries. This reinforces the robustness of our model in describing the complex dynamics of microfluidic synthesis of mesoporous hollow silica microcapsules. This model is experimentally supported by SEM images showing droplets in the midst of division (Figure 5). The observed splitting illustrates the physical limitations on shell thickness and density, reinforcing the need to consider multiple spheres per droplet in modeling the synthesis process.

An alternative strategy to obtain larger microcapsules involves merging smaller ones during the early stage of synthesis. By collecting small, partially solidified droplets in a common reservoir and carefully controlling the microfluidic flow conditions and surfactant concentration, the likelihood of microcapsule contact and merging is enhanced. The shell material, which remains in a gel-like or liquid state at this stage, enables the boundaries of adjacent microcapsules to merge, resulting in the formation of a single, larger spherical capsule. This merging technique is well-established in emulsion-based microcapsule synthesis and microfluidic fabrication, providing a simple yet effective route to larger microcapsules beyond the size limitations of single droplet formation [36]. Rather than the merging of liquid droplets, coalescence in our experiments occurs through the merging of already-formed small microspheres. This merging typically arises due to geometric constraints within the microfluidic reservoir or channel such as packing density, channel width, or hydrodynamic flow patterns as well as factors like surfactant concentration and other fluid dynamic interactions. By reducing the surfactant concentration, it is possible to promote coalescence before the capsules become fully rigid. Multiple pre-formed microspheres may come into adhesive contact and unite into a single, larger capsule, with traces of the initial microspheres sometimes visible on the final shell structure. However, the occurrence of such merging is largely governed by local conditions and chance, and cannot be precisely controlled under the present synthesis protocol. This phenomenon is fundamentally distinct from single-droplet templating followed by growth.

**Figure 6:**
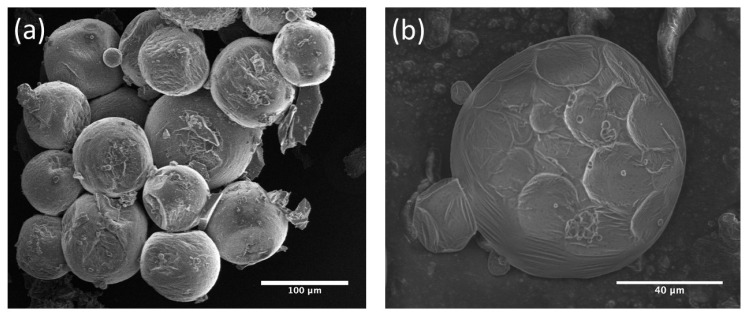
SEM images of (**a**) different sizes of microspheres obtained by coalescence. (**b**) Resulting large microsphere from the merging of several smaller microspheres (Final diameter 88 μm).

Experimentally, we observed that this approach occasionally produced microcapsules with diameters in the range of 40–90 µm (Figure 6a), far exceeding those predicted by the original 10 µm capsule geometry. SEM images revealed that coalescence typically involved the fusion of multiple primary droplets, as evidenced by the discernible traces of the initial spheres on the final capsule shell in Figure 6b. The final resulting microcapsules remain highly spherical with smooth, continuous shells, indicating that the merging process does not disrupt mesostructure formation.

This synthesis pathway thus provides an effective, flexible route for obtaining larger microresonators without modifying either the microfluidic channel geometry or the TEOS concentration. Such larger capsules are critical for this study, as they offer longer optical path lengths and higher refractive index contrast, enabling clear observation of whispering gallery modes.

### 3.2. Observation of Whispering Gallery Modes in Mesoporous Hollow Silica Capsules

Fluorescence emission spectra obtained from Rhodamine B–loaded mesoporous hollow silica microcapsules reveal the clear presence of whispering gallery modes (WGMs). Upon 532 nm excitation, the dye emits a broad luminescent background, on which a periodic train of sharp spectral peaks is superimposed—characteristic of resonant light confinement within a spherical geometry.

**Figure 7:**
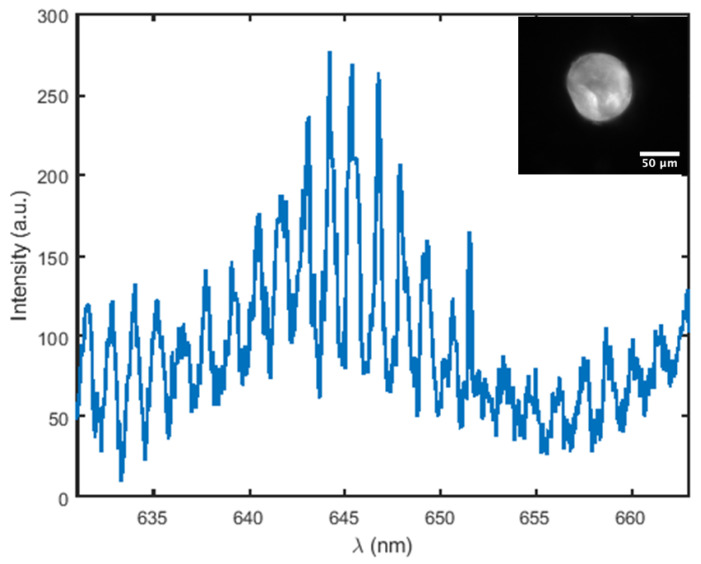
Fluorescence spectrum and image from a Rhodamine B–doped mesoporous silica capsule (radius 44 µm). Narrow, periodic peaks superimposed on the dye background indicate the presence of high-quality whispering gallery modes. The corresponding quality factors of the resolved resonances range from Q  ≈  600 to Q  ≈  2700.

Figure 7 displays a representative spectrum from a single capsule with radius R = 44μm. The observed free spectral range (FSR), i.e., the intermodal peak spacing, is Δλ = 1.296nm. The peaks are narrow and evenly spaced, with high contrast against the fluorescent background, indicating strong optical feedback and minimal scattering losses within the structure.

The emergence of WGMs in these microcapsules is a nontrivial observation. Despite the shell’s nanoporosity, which typically leads to scattering and index fluctuations, the capsules support well-defined optical modes. This confirms that the radial symmetry, smooth shell surface, and uniform porosity achieved via droplet-based microfluidic synthesis are sufficient to maintain high optical quality. The formation of WGMs is governed by the resonance condition for a given positive integer mode number m:

$$2\pi Rn_{\mathrm{eff}}\;=\;m\lambda_{m}$$

where *R* is the capsule radius, $n_{\mathrm{eff}}$ is the effective refractive index of the shell, $\lambda_{m}$ is the resonance wavelength, and *m* is the mode number. The observed periodicity in wavelength directly encodes the optical path length within the shell, enabling model-free estimation of $n_{\mathrm{eff}}$.

This result validates the use of mesoporous hollow silica microcapsules as functional WGM resonators and opens the door to extracting quantitative optical parameters from their emission spectra. In particular, the spectral spacing Δλ provides direct access to the effective refractive index, which in turn reflects the shell’s nanoscale porosity. This approach enables non-destructive, single-particle refractometry of mesostructured dielectric materials—something previously accessible only via ensemble-averaged techniques such as BET or ellipsometry. This initial spectral observation is more than a proof of concept; it is a gateway to a broader optical metrology framework for nanoporous materials.

To justify the identification of the observed spectral resonances as whispering gallery modes, we evaluated the quality factor (Q  =  λ/Δλ) of each resolved peak in the photoluminescence spectrum shown in Figure 7. The extracted quality factors range from $Q\;\approx \;6\times 10^{2}$ up to $Q\;\approx \;2.7\times 10^{3}$ across the measured wavelength interval. We emphasize that, due to the strong photoluminescence background, finite spectrometer resolution, and the pulsed excitation regime, these values should be regarded as effective or apparent *Q*-factors rather than intrinsic cavity quality factors. Similar apparent high-*Q* features may arise in other optical systems when measured in emission. In the present work, *Q*-factors are therefore used only as an order-of-magnitude indicator of resonant confinement and not as a central quantitative parameter.

Such values are fully consistent with whispering gallery modes in mesoporous silica microresonators operating under photoluminescence excitation, where optical losses are dominated by material absorption, surface scattering, and radiation leakage [22,37]. In contrast, non-resonant spherical modes or leaky cavity states typically exhibit significantly lower quality factors and do not produce the quasi-periodic spectral structure observed experimentally.

Moreover, although individual resonances may appear broadened due to ensemble effects and finite spectrometer resolution, the persistence of a well-defined free spectral range over multiple consecutive peaks confirms the presence of circulating optical modes confined by total internal reflection. This collective behavior is a defining signature of whispering gallery modes and cannot be attributed to random photoluminescence features or low-order Mie resonances.

We therefore emphasize that the analysis presented in this work does not rely on isolated high-*Q* resonances, but rather on the global modal periodicity extracted through Fourier-transform analysis, which is intrinsically robust to moderate *Q* variations and mode broadening.

### 3.3. Refractive Index Extraction via Fourier Transform Analysis

To quantitatively extract the effective refractive index $n_{\mathrm{eff}}$ of the mesoporous silica shell, we applied Fourier Transform (FT) analysis to the fluorescence spectrum of an individual Rhodamine B-loaded capsule. This method probes the optical path length traversed by whispering gallery modes (WGMs), which are sensitive to both the geometric and dielectric properties of the resonator.

Prior to FT analysis, the experimentally recorded fluorescence spectra were systematically preprocessed to ensure robust extraction of the WGM periodicity and to minimize artifacts related to background emission and finite spectral windows. Raw spectra consist of a broadband Rhodamine B fluorescence background with superimposed resonant modulations arising from optical cavity modes. First, the recorded intensity was normalized to the [0, 1] interval after subtracting the minimum value of the spectrum, which suppresses detector offsets and enables consistent comparison between different measurements. For each identified WGM mode group, a restricted wavelength interval was selected to isolate a quasi-periodic spectral region with approximately constant free spectral range (FSR), thereby avoiding artificial broadening of the Fourier peaks caused by slow dispersion over wide spectral windows. Within each selected interval, the mean intensity was subtracted to remove the slowly varying fluorescence background and suppress the zero-frequency component in the Fourier spectrum. A Hann apodization window was then applied prior to computing the fast Fourier transform, which minimizes spectral leakage due to the finite acquisition window and improves localization of the dominant Fourier peak associated with the WGM optical path length.

The modulus of the Fourier transform was symmetrized and normalized to its maximum value, and the Fourier axis was converted from inverse-wavelength units to optical path length using the central wavelength of the selected spectral region. Finally, the effective refractive index was obtained by rescaling the optical path length by the measured capsule radius according to the WGM resonance condition.

The periodicity of the WGM peaks was determined from the FT amplitude spectrum as Δλ = 1.296 nm (average value over the spectral window 635–660 nm). Although the FSR exhibits a slight dispersion in wavelength space, the Fourier method yields a robust mean estimate. Using the central WGM resonance wavelength λ = 645 nm, we calculated the optical path length as:

$$l_{\mathrm{opt}}\;=\;\frac{\lambda^{2}}{\Delta \lambda }\;\approx \;321.2\;\mu m$$

For a capsule radius R = 44μm This yielded an effective refractive index of:

$$n_{\mathrm{eff}}\;=\;\frac{l_{\mathrm{opt}}}{2\pi R}\;\approx \;1.164$$

**Figure 8:**
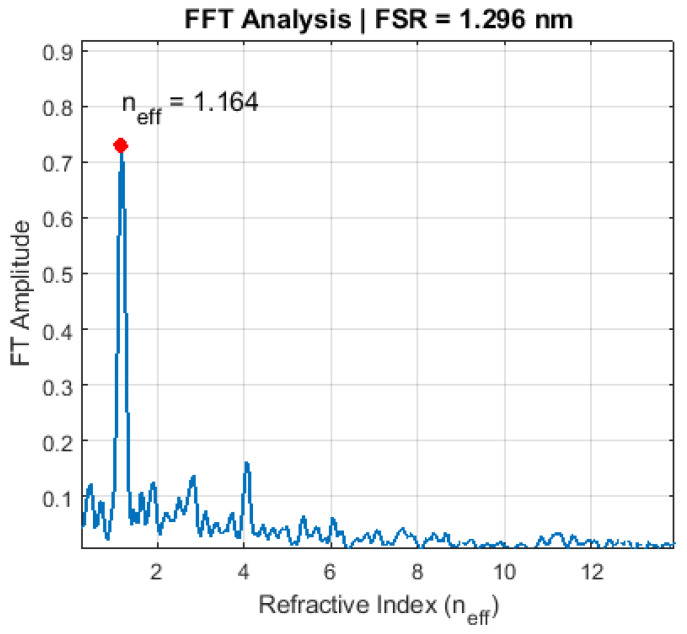
Fourier transform amplitude spectrum (after preprocessing) rescaled using $n_{\mathrm{eff}}\;=\;l_{\mathrm{opt}}/\left(2\pi R\right)$. The dominant peak confirms $n_{\mathrm{eff}}\;=\;1.164$ from the WGM optical path length $l_{\mathrm{opt}}\;\approx \;321.2\;\mu m$.

The dominant peak in the FT amplitude spectrum (Figure 8) confirms this value. The method bypasses direct peak indexing, providing robust sensitivity to sub-wavelength dielectric changes.

The uncertainty associated with the extracted effective refractive index arises from three independent experimental contributions: (i) the determination of the central wavelength λ, (ii) the extraction of the intermodal spacing Δλ from the Fourier transform, and (iii) the measurement of the capsule radius *R*. Error propagation was evaluated assuming these contributions to be uncorrelated.

The relative uncertainty on the effective refractive index is given by

(1) $${\left(\frac{\delta n_{\mathrm{eff}}}{n_{\mathrm{eff}}}\right)}^{2}\;=\;{\left(2\;\frac{\delta \lambda }{\lambda }\right)}^{2}+{\left(\frac{\delta (\Delta \lambda )}{\Delta \lambda }\right)}^{2}+{\left(\frac{\delta R}{R}\right)}^{2},$$

where λ is the mean resonance wavelength of the analyzed spectral window. Using representative values δλ∼0.1 nm, δ(Δλ)∼0.1 nm, and δR/R  ≈  1–2%, this yields:

$$n_{\mathrm{eff}}\;\;=\;\;1.164\pm 0.07$$

The wavelength uncertainty δλ is set by the spectrometer calibration and spectral resolution (typically ∼0.1 nm), while δ(Δλ) reflects the finite width of the dominant peak in the Fourier amplitude spectrum and was conservatively estimated from its full width at half maximum. In practice, the Fourier-based determination of Δλ benefits from averaging over multiple resonances within each spectral window, which significantly reduces sensitivity to noise and individual peak broadening.

The dominant contribution to the overall uncertainty originates from the capsule radius determination (δR/R ≈ 1–2%), which is limited by optical microscopy calibration and the finite depth of field for large microcapsules. Consequently, the reported uncertainty represents a conservative upper bound rather than an intrinsic limitation of the Fourier-transform-based WGM analysis.

Despite this conservative estimate, the extracted effective refractive index leads to porosity values that agree within less than 1% with independent Brunauer–Emmett–Teller (BET) measurements. This agreement confirms the robustness of the method and demonstrates that the principal source of uncertainty is geometric rather than spectroscopic in origin.

This low effective index ($n_{\mathrm{eff}}\ll n_{{\mathrm{SiO}}_{2}}\;=\;1.47$) reflects the shell’s high porosity. The FT approach enables precise refractometry even in noisy spectra, outperforming bulk-averaging methods.

### 3.4. Porosity Determination via Effective Medium Models

To quantitatively relate the measured effective refractive index ($n_{\mathrm{eff}}\;=\;1.164$) to the shell’s porosity (*p*), we employed classical effective medium approximations (EMAs). The mesoporous silica is treated as a binary composite of dense silica ($n_{{\mathrm{SiO}}_{2}}\;=\;1.47$) and air-filled pores ($n_{\mathrm{air}}\;=\;1.00$). We compare three widely used models, each based on distinct assumptions about pore geometry and connectivity:

1. **Lorenz–Lorentz (LL)**: Assumes spherical pores in a continuous silica matrix, derived from the Clausius–Mossotti relation for dilute systems:

$$\frac{n_{\mathrm{eff}}^{2}-1}{n_{\mathrm{eff}}^{2}+2}\;=\;\left(1-p\right)\frac{n_{{\mathrm{SiO}}_{2}}^{2}-1}{n_{{\mathrm{SiO}}_{2}}^{2}+2}$$

yielding p = 0.620 (62.0%).

2. **Bruggeman**: Treats both phases symmetrically, appropriate for interconnected or bicontinuous porous networks:

$$\left(1-p\right)\frac{n_{{\mathrm{SiO}}_{2}}^{2}-n_{\mathrm{eff}}^{2}}{n_{{\mathrm{SiO}}_{2}}^{2}+2n_{\mathrm{eff}}^{2}}+p\frac{1-n_{\mathrm{eff}}^{2}}{1+2n_{\mathrm{eff}}^{2}}\;=\;0$$

Numerical solution gives p = 0.633 (63.3%).

3. **Maxwell–Garnett (MG)**: Describes spherical, non-interacting air inclusions in a continuous silica matrix:

$$\frac{\epsilon_{\mathrm{eff}}-\epsilon_{m}}{\epsilon_{\mathrm{eff}}+2\epsilon_{m}}\;=\;p\frac{\epsilon_{i}-\epsilon_{m}}{\epsilon_{i}+2\epsilon_{m}}$$

where $\epsilon_{\mathrm{eff}}\;=\;n_{\mathrm{eff}}^{2}$, $\epsilon_{m}\;=\;n_{{\mathrm{SiO}}_{2}}^{2}$, and $\epsilon_{i}\;=\;1$. Solving yields p = 0.650 (65.0%).

**Figure 9:**
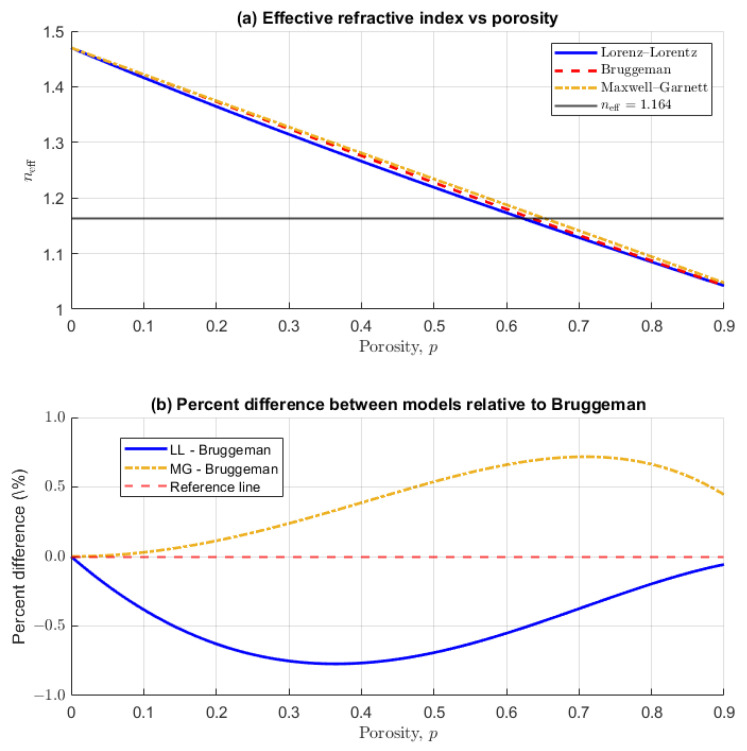
Effective refractive index versus porosity for mesoporous silica, comparing three effective medium approximations. (**a**) Lorenz–Lorentz (blue solid), Bruggeman (red dashed), and Maxwell–Garnett (gold dotted). The horizontal line at $n_{\mathrm{eff}}\;=\;1.164$ shows how model assumptions affect porosity predictions. (**b**) Percent difference between models relative to Bruggeman. Note the divergence of Maxwell–Garnett at higher porosities.

The comparison of these models reveals important insights into pore structure (Figure 9 and Table 2): while Lorenz–Lorentz and Bruggeman predictions closely bracket the BET reference value of 62.8%, Maxwell–Garnett yields a significantly higher porosity of 65.0%. This 2.2% discrepancy relative to BET is physically informative: the Maxwell–Garnett model assumes isolated, non-interacting spherical pores, whereas our mesoporous silica exhibits interconnected porosity as evidenced by nitrogen adsorption hysteresis. The Bruggeman model, designed for symmetric, interconnected mixtures, provides the closest match to BET (Δ = +0.5%), supporting the characterization of our material as having a bicontinuous pore network.

The relative positions of the EMA curves (Figure 9) reveal their underlying geometric assumptions. At low porosities (p<0.3), Maxwell–Garnett and Bruggeman converge because both effectively model air inclusions in a continuous silica matrix—MG explicitly assumes spherical inclusions, while Bruggeman’s symmetric form approximates this limit when one phase is dilute. Conversely, at high porosities (p>0.7), Lorenz–Lorentz and Bruggeman converge as both describe silica inclusions in an air matrix. This crossover highlights Bruggeman’s role as a symmetric interpolant between asymmetric extremes.

Mathematically, the convergence of LL and Bruggeman predictions (Δ<1.3%) despite their different geometric assumptions can be understood by examining their equations. For a binary composite where one phase has dielectric constant $\epsilon_{i}\;\approx \;1$ (air), both models reduce to similar functional forms. The Bruggeman equation:

$$\left(1-p\right)\frac{\epsilon_{m}-\epsilon_{\mathrm{eff}}}{\epsilon_{m}+2\epsilon_{\mathrm{eff}}}+p\frac{1-\epsilon_{\mathrm{eff}}}{1+2\epsilon_{\mathrm{eff}}}\;=\;0$$

and the Lorenz–Lorentz relation:

$$\frac{\epsilon_{\mathrm{eff}}-1}{\epsilon_{\mathrm{eff}}+2}\;=\;\left(1-p\right)\frac{\epsilon_{m}-1}{\epsilon_{m}+2}$$

yield nearly identical solutions when $\epsilon_{m}$ is small ($\epsilon_{{\mathrm{SiO}}_{2}}\;=\;2.16$). The difference scales approximately as ${\left(\epsilon_{m}-1\right)}^{2}$, which for silica is only ∼1.35. This explains why geometric corrections remain minimal: at low dielectric contrast, field perturbations around pores are weakly dependent on pore shape and connectivity.

This contrasts with high-index materials like titanium dioxide (n ≈ 2.5, ϵ ≈ 6.25), where the same porosity would produce a >5% discrepancy between LL and Bruggeman predictions. Our silica/air system thus occupies a fortuitous regime where model choice has negligible impact on porosity extraction. The inclusion of Maxwell–Garnett further contextualizes this analysis: while LL and Bruggeman bracket the BET reference (62.0–63.3%), MG’s assumption of isolated spherical pores overestimates porosity by 2.2% (65.0%), supporting the characterization of our material as having interconnected porosity. The modest contrast of silica/air ($\epsilon_{m}/\epsilon_{i}\;=\;2.16$) thus explains both the LL-Bruggeman convergence and the relatively small MG deviation; in higher-contrast systems, all three models would diverge significantly, making geometric assumptions critical.

The excellent agreement between Bruggeman-predicted porosity (63.3%) and BET (62.8%), as reported in Table 2, confirms several key points:

- Single-particle sensitivity: WGM spectroscopy accurately captures the effective optical porosity of individual microcapsules, avoiding ensemble averaging inherent to bulk techniques.
- Pore connectivity: The superior performance of Bruggeman over Maxwell–Garnett corroborates the interconnected nature of the mesoporous network, consistent with type H1 hysteresis observed in adsorption isotherms for P123-templated materials.
- Architectural confirmation: The high porosity (∼63%) quantitatively validates the hollow-core, radially ordered mesoporous shell architecture achieved via microfluidic synthesis.

This analysis establishes WGM spectroscopy combined with appropriate effective medium modeling as a powerful, non-destructive technique for porosity quantification at the single-particle level. The method’s micron-scale spatial resolution enables probing of local porosity variations in hierarchically structured materials, offering complementary insights to ensemble-averaged gas adsorption techniques.

### 3.5. Limitations

While the present work demonstrates the feasibility and accuracy of whispering gallery mode (WGM) spectroscopy for single-particle refractive index and porosity measurements in mesoporous hollow silica microcapsules, several limitations should be acknowledged.

First, the analysis assumes a perfectly spherical geometry with uniform shell thickness. In practice, small deviations in roundness or shell uniformity may introduce systematic shifts in the extracted $n_{\mathrm{eff}}$. However, the Fourier transform-based approach remains robust to moderate imperfections: it extracts the average optical path length rather than relying on precise resonance shapes. For ellipsoids with aspect ratios up to ∼1.1, resonance broadening remains smaller than the free spectral range (FSR), allowing reliable peak discrimination. Our observed *Q*-factors of ∼$10^{3}$ and SEM images (Figure 2) confirm high sphericity. Beyond aspect ratios of ∼1.2, peak overlap can compromise FSR extraction, requiring alternative approaches such as Mie theory fitting or digital holography.

Second, the method probes only one particle at a time; while this single-particle sensitivity is advantageous for heterogeneity studies, it means statistical variability across populations requires multiple measurements.

Third, porosity determination relies on effective medium approximations (Lorenz–Lorentz, Bruggeman) that assume simplified pore shapes and connectivity, which may not fully reflect the complexity of the actual mesoporous network. The close agreement with BET measurements validates these approximations for our system but may require adjustment for other pore geometries.

Fourth, the measurement is performed at a single fluorescence wavelength (λ ≈ 645 nm), without accounting for potential dispersion of $n_{\mathrm{eff}}$ across the visible or near-infrared spectrum.

Finally, all measurements were conducted in air on dried capsules; the influence of surrounding media (e.g., liquids, vapors) or dynamic infiltration processes on WGM spectra remains to be explored. Addressing these aspects in future work would further strengthen the robustness and applicability of the technique to a wider range of mesostructured materials and operating conditions.

## 4. Conclusions

This study demonstrates that mesoporous hollow silica microcapsules, fabricated via microfluidic emulsion templating, can function as high-quality optical microresonators supporting whispering gallery modes (WGMs) despite their high porosity. By incorporating Rhodamine B fluorophores into the capsule shells and applying a refined Fourier Transform (FT) analysis of the fluorescence spectra, we determined the effective refractive index of individual capsules with high precision ($n_{\mathrm{eff}}\;=\;1.164\pm 0.07$). The measured intermodal spacing (Δλ = 1.296 nm) corresponds to an optical path length of 321.2 µm, consistent with the hollow-core, radially ordered mesoporous architecture obtained from microfluidic synthesis.

Using three classical effective medium approximations—Lorenz–Lorentz, Bruggeman, and Maxwell–Garnett—the WGM-derived refractive index translated to porosities of 62.0%, 63.3%, and 65.0%, respectively. The close agreement between the Bruggeman prediction (63.3%) and the Brunauer–Emmett–Teller (BET) reference value (62.8%) confirms that WGM spectroscopy provides a robust, non-destructive alternative to gas adsorption techniques, with the added advantage of single-particle sensitivity and micron-scale spatial resolution. Moreover, the model comparison yields physical insight: Bruggeman’s superior performance corroborates an interconnected pore network, whereas Maxwell–Garnett’s overestimate reflects its assumption of isolated spherical pores.

Beyond validating the methodology, this work establishes a direct optical link between nanoscale porosity and macroscopic refractive index in mesostructured silica. The ability to interrogate individual microcapsules opens new possibilities for in situ refractometric sensing, monitoring of infiltration dynamics, and optical quality control of porous photonic building blocks. The approach is readily adaptable to other nanostructured dielectrics and could be extended to liquid-phase measurements or dispersive wavelength regimes to further enhance its applicability.

By bridging microfluidic synthesis, mesoporous material engineering, and cavity-based optical metrology, this work provides both a methodological framework and a proof-of-concept for high-sensitivity, single-particle refractometry in advanced porous materials.

## Figures and Tables

**Table 1:** Dependence of hollow silica microcapsules size on droplet size, including experimentally measured shell thickness, theoretical shell thickness, and estimated number of final spheres per droplet.

$d_{\mathrm{drop}}$ (µm)	$d_{\mathrm{part}}$ (µm)	$h_{\mathrm{shell},\exp}$ (µm)	$h_{\mathrm{shell},\mathrm{theor}}$ (µm)	Number of Daughters *n*
30 ± 0.9	10 ± 0.5	1.1 ± 0.17	1.1 ± 0.17	1
50 ± 1.5	10 ± 0.5	1.3 ± 0.20	1.28 ± 0.19	4
70 ± 2.1	10 ± 0.5	1.5 ± 0.23	1.76 ± 0.26	8
90 ± 2.7	20 ± 1.0	1.8 ± 0.27	1.86 ± 0.28	4
120 ± 3.6	22 ± 1.1	2.2 ± 0.33	2.43 ± 0.36	6
130 ± 3.9	30 ± 1.5	2.5 ± 0.38	2.5 ± 0.38	4

**Table 2:** Porosity values derived from WGM spectroscopy using different effective medium models, compared with BET measurements.

Method	Porosity (*p*)	Deviation from BET
WGM (Lorenz—Lorentz)	62.0%	−0.8%
WGM (Bruggeman)	63.3%	+0.5%
WGM (Maxwell–Garnett)	65.0%	+2.2%
BET (reference)	62.8%	–

## Data Availability

The original contributions presented in this study are included in the article. Further inquiries can be directed to the corresponding author.
